# Concentrations of S100B and neurofilament light chain in blood as biomarkers for checkpoint inhibitor–induced CNS inflammation

**DOI:** 10.1016/j.ebiom.2023.104955

**Published:** 2024-01-03

**Authors:** Sara Bjursten, Zhiyuan Zhao, Hifaa Al Remawi, Marie Studahl, Ankur Pandita, Joel Simrén, Henrik Zetterberg, Anna-Carin Lundell, Anna Rudin, Lars Ny, Max Levin

**Affiliations:** a Department of Oncology, Institute of Clinical Sciences, Sahlgrenska Academy at University of Gothenburg, Sweden; b Department of Oncology, Sahlgrenska University Hospital, Gothenburg, Sweden; c Department of Molecular and Clinical Medicine/Wallenberg Laboratory, Institute of Medicine, Sahlgrenska Academy, University of Gothenburg, Gothenburg, Sweden; d Department of Infectious Diseases, Institute of Biomedicine, Sahlgrenska Academy, University of Gothenburg, Gothenburg, Sweden; e Department of Infectious Diseases, Sahlgrenska University Hospital, Gothenburg, Sweden; f Department of Psychiatry and Neurochemistry, Institute of Neuroscience and Physiology, The Sahlgrenska Academy at the University of Gothenburg, Mölndal, Sweden; g Clinical Neurochemistry Laboratory, Sahlgrenska University Hospital, Mölndal, Sweden; h Department of Neurodegenerative Disease, UCL Institute of Neurology, Queen Square, London, UK; i UK Dementia Research Institute at UCL, London, UK; j Hong Kong Center for Neurodegenerative Diseases, Hong Kong, China; k Department of Rheumatology and Inflammation Research, Institute of Medicine, Sahlgrenska Academy, University of Gothenburg, Gothenburg, Sweden

**Keywords:** Checkpoint inhibitors, Immune related adverse events of the CNS, Brain damage markers, S100B, Neurofilament light, Incidence

## Abstract

**Background:**

Cancer treatment with immune checkpoint inhibition (ICI) can cause immune-related adverse events in the central nervous system (CNS irAE). There are no blood biomarkers to detect CNS irAE. We investigated if concentrations of S100-calcium-binding protein B (S100B) and neurofilament light chain (NfL) in blood can be used as biomarkers for CNS irAE and assessed the incidence of CNS irAE in a cohort of ICI-treated patients.

**Methods:**

In this single-centre, retrospective cohort study, we examined medical records and laboratory data of 197 consecutive patients treated with combined CTLA-4 and PD-1 inhibition (ipilimumab; ipi + nivolumab; nivo) for metastatic melanoma or renal cell carcinoma. CNS irAE was diagnosed using established criteria. Concentrations of S100B and NfL in blood were measured in patients with CNS irAE and in 84 patients without CNS irAE.

**Findings:**

Nine of 197 patients (4.6%) fulfilled criteria for CNS irAE. S100B and NfL in blood increased during CNS inflammation and normalized during immunosuppression. CNS irAE was detected with a sensitivity of 100% (S100B) and 79% (NfL) and a specificity of 89% (S100B) and 74% (NfL). Patients with CNS irAE had simultaneous increased concentration of C-reactive protein (CRP) (9/9) and alanine aminotransferase (ALT) and/or aspartate aminotransferase (AST) in blood (8/9).

**Interpretation:**

Analysis of S100B, NfL and CRP in blood facilitates the diagnosis of CNS irAE. CNS irAE may be more common than previously reported. There may be shared immune mechanisms between CNS and hepatitis irAE.

**Funding:**

Supported by funding from the 10.13039/100012538Swedish Cancer Foundation, the ALF-agreement, and Jubileumsklinikens Cancerfond.

**Research in context**

**Evidence before this study**

Cancer treatment with immune checkpoint inhibitors can cause immune-related adverse events (irAE). IrAE range in severity from mild to lethal and can affect any organ, including the brain (CNS irAE). Blood tests are invaluable tools for the early detection of most irAE but there are currently no available blood tests to detect CNS irAE. Previous studies have shown that the risk for CNS irAE is higher in patients treated with combined PD-1 and CTLA-4 inhibition (nivolumab; nivo + ipilimumab; ipi) than in patients treated with single PD-1 or PD-L1 inhibition. The reported frequencies of CNS irAE after double checkpoint inhibition (ipi + nivo) vary from less than 1% in clinical trials to 2.8% in retrospective studies.

**Added value of this study**

In this retrospective cohort study of 197 patients treated with ipi + nivo, we demonstrate that analysis of brain damage markers S100B and neurofilament light chain (NfL) in blood has a high sensitivity and specificity for early detection and monitoring of CNS irAE. Additionally, the incidence of CNS irAE in our cohort was 4.6% which is higher than previously reported, suggesting that CNS irAE may be underdiagnosed.

**Implications of all the available evidence**

Analysis of S100B and NfL in blood in patients treated with ipi + nivo could facilitate diagnosis of checkpoint inhibitor induced CNS irAE.

## Introduction

Immune checkpoint inhibition (ICI) improves survival in a growing number of cancers.^1^, ^2^, ^3^ ICI activates T cells by blocking two inhibitory T cell receptors: programmed cell death protein 1 (PD-1) with nivolumab (nivo) or pembrolizumab (pembro) and cytotoxic T-lymphocyte-associated protein 4 (CTLA-4) with ipilimumab (ipi). Activation increases the capacity of T cells to eradicate cancer cells but may also cause autoimmune and inflammatory toxicities.^4^, ^5^, ^6^ These immune-related adverse events (irAE) may affect any organ and range in severity from mild to deadly. ICI–induced inflammation in the central nervous system (CNS irAE), causing encephalitis or aseptic meningitis, is a rare but serious irAE.^7^, ^8^, ^9^, ^10^, ^11^ The risk of CNS irAE is higher with ipi + nivo (simultaneous blockade of CTLA-4 and PD-1) than with single nivo (PD-1 inhibition).^12^

CNS irAE is difficult to diagnose because the symptoms are often nonspecific (confusion, altered behaviour, light sensitivity, headache) or suggest more common conditions, such as tumour progression (paresis) or infection (high fever).^8^^,^^10^^,^^11^ Moreover, symptoms often appear weeks or months after ICI treatment, and patients may seek care in the emergency room or at local health care centres, rather than at an oncology clinic. As a result, the correct diagnosis and necessary immunosuppressive treatment may be delayed, increasing risk for severe neurological sequelae or even death. Therefore, simple tests to facilitate the diagnosis of CNS irAE are needed.

Blood tests are invaluable tools for the early detection of most irAE but there are currently no available blood tests to detect CNS irAE.^4^, ^5^, ^6^ We recently discovered high concentrations of two brain damage markers in blood—S100 calcium-binding protein B (S100B) and neurofilament light (NfL)—in a patient with severe CNS irAE.^8^ S100B is released by damaged astrocytes and is used in clinical routine as a marker of traumatic brain injury.^13^ In addition, serum S100B is also used to monitor metastatic melanoma because melanoma metastases produce S100B in some, but not all, patients with melanoma.^14^ NfL is increasingly used as a brain damage marker in a wide range of conditions including neuroinflammatory diseases,^15^, ^16^, ^17^, ^18^ traumatic brain injury^19^ and after brain surgery.^20^ Therefore, we investigated increased concentrations of S100B and NfL in blood as biomarkers for CNS irAE. We also determined the incidence of CNS irAE in a cohort of patients treated with ipi + nivo.

## Methods

### Patient cohort

All patients with metastatic melanoma (n = 144) or metastatic renal cell carcinoma (n = 53) treated with ipi + nivo between March 2018 and April 2022 at the Oncology Department, Sahlgrenska University Hospital, were included in the study. Patients with melanoma received either ipi 3 mg/kg + nivo 1 mg/kg or ipi 1 mg/kg + nivo 3 mg/kg. Patients with renal cell carcinoma received ipi 1 mg/kg + nivo 3 mg/kg. Treatment was given up to 4 times at 3-week intervals followed by nivolumab monotherapy. Adverse events were documented in the patients’ electronic medical records. A standardized panel of blood tests to detect irAE were analysed repeatedly during ICI treatment. The panel included blood electrolytes, blood cell counts, creatinine, aspartate aminotransferase (AST), alanine aminotransferase (ALT), bilirubin, alkaline phosphatase (ALP), creatinine kinase (CK), thyroid stimulating hormone (TSH), thyroxine, cortisol, C-reactive protein (CRP; a marker of systemic inflammation) and S100B (melanoma marker).

### Cases with CNS irAE

Patients with suspected CNS irAE underwent magnetic resonance imaging of the brain and a lumbar puncture. Three consensus criteria were used to diagnose CNS irAE: excluding tumour progression or infection (1), CNS symptoms that improve with immunomodulation (2), pleocytosis (>5 white blood cells/μl) and/or elevated protein in cerebrospinal fluid (CSF) (3).^21^ The consensus criteria for CNS irAE are modified from clinical criteria to diagnose autoimmune encephalitis.^22^ CSF was also analysed for the brain damage markers NfL, glial fibrillary acidic protein (GFAP), and tau^23^^,^^24^ and for neuronal antibodies, interleukins, and cytokines.

### Patients without CNS irAE

S100B is a marker of both melanoma and brain damage, as it is secreted by some melanoma metastases and by damaged astrocytes.^14^^,^^25^ Because it is a melanoma marker, serum S100B was analysed monthly during treatment in all patients with melanoma (n = 144), including those that developed CNS irAE. Patients with melanoma who did not develop CNS irAE and whose metastases were S100B-negative (n = 29) and patients with renal cell carcinoma (n = 10) served as ipi + nivo–treated controls for the S100B analysis (Supplmentary Table S1). S100B peaks in patients with CNS irAE were compared to the highest S-100B concentration obtained within 200 days (from first ipi + nivo) in patients without CNS irAE. Plasma NfL was analysed in patients with suspected CNS irAE. The plasma NfL concentrations of controls—patients with melanoma treated with either ipi + nivo or single PD1 inhibitor, nivo or pembro, who did not develop CNS irAE (n = 49) —were determined in frozen plasma samples (Supplmentary Table S2).

### Cerebrospinal fluid from patients with herpes simplex virus encephalitis

CSF from patients with herpes simplex encephalitis were included in the study (n = 8). The patients had previously been included in prospective studies of herpes simplex encephalitis at the Department of Infectious Diseases, Sahlgrenska University Hospital, Gothenburg, Sweden. Patients had clinical signs of encephalitis and HSV-1 DNA detected in CSF by PCR. The CSF samples were drawn within a month after onset of neurological symptoms, in mean 13 days, median 13, range 0–29 days. The CSF samples had been stored frozen in −80 °C.

### Cerebrospinal fluid from SLE patients without CNS inflammation

Cerebrospinal fluid samples (CSF) from patients with systemic lupus erythematosus without symptoms of encephalitis or meningitis were included as controls (n = 16). The CSF samples had been stored frozen in −80 °C.

### Research ethics

Retrospective analysis of the patients’ electronic medical records was approved by the Regional Ethics Board in Gothenburg (477-18, 2021-04093). Frozen plasma samples from a set of patients were used for retrospective analysis of NfL. This analysis was also approved by the Regional Ethics Board (151-16), and written informed consent was obtained from all participants. Ethical approval for analysis of CSF from patients with virus encephalitis was obtained from the Regional Ethics Board at Gothenburg University (229-14 and 402-18) and the Regional Ethics Board at Karolinska Institute (99–409 and amendment 03–380). Analysis of cerebrospinal fluid from patients with autoimmune systemic lupus erythematosus was approved by the Regional Ethics Board at Gothenburg University (433-11).

### Analysis of S100B, NfL, GFAP and tau

Serum S100B concentrations were measured with a commercially available electrochemiluminescence immunoassay on a Cobas platform (Roche Diagnostics, Penzberg, Germany). Plasma NfL concentration was measured with a single-molecule-array assay on an HD-X Analyzer (Quanterix, Billerica, MA). CSF concentrations of NFL and glial fibrillary acidic protein (GFAP) were measured with in-house enzyme-linked immunosorbent assays as described.^26^^,^^27^ CSF tau concentration was measured with a Lumipulse immunoassay.^28^

### Analysis of neuronal autoantibodies

Plasma and serum from patients with CNS irAE were analysed for neuronal autoantibodies directed to intracellular paraneoplastic antigens and surface antigens by the Clinical Immunology Laboratory at Sahlgrenska University Hospital (accredited by Swedac). The methodology included indirect immune fluorescence mosaics with sections of primate cerebellum/intestine (Biochip–Neurology mosaic 14) or transfected cells (Biochip—Autoimmune encephalitis mosaic 6) and confirmation with immunoblot (Euroline-Paraneoplastic Neurologic Syndromes) according to manufactures instruction. Anti-GAD 65 antibodies were analysed by ELISA, Anti-GAD ELISA (IgG). All products were from Euroimmun Medizinische Labordiagnostika AG (Lübeck, Germany).

### Statistics

Lymphocytes and NfL in CSF and CSF/serum albumin quotients in patients with CNS irAE, herpes simplex encephalitis and controls without CNS inflammation were compared with Kruskal–Wallis test. Peak values of S100B, NfL, CRP and ALT in patients with or without CNS irAE were compared with the Mann–Whitney U test. Concentrations in blood of S100B, NfL, CRP and ALT during active and resolved CNS irAE were compared with the two-tailed sign test. The sensitivity and specificity of concentrations in blood of S100B, NfL, CRP and ALT for CNS irAE were calculated by using receiver operating characteristic curves; the cut-off point was the upper limit of normal. Graph Pad Prism v9.3.1 was used for statistical analyses and for creating graphs. P-values of <0.05 were considered statistically significant. Two-tailed sign test was performed using Excel with XLSTAT add-on (Lumivero). Quantitative variables for CNS irAE patients included age, time in hospital, time to CNS irAE, and follow-up time. These variables are presented as mean with standard deviation (SD) for symmetrical data and median with interquartile range (IQR) for asymmetrical data. The sample size was all female and male patients treated with ipi + nivo at one single centre during a 50-month period (March 2018 and April 2022).

### Role of the funders

The funding sources had no role in study design, collection of data, analysis and interpretation of data, in writing the report, or in the decision to submit the paper for publication.

## Results

### Diagnosis and characteristics of CNS irAE

Nine (4.6%) of 197 patients (five women and four men) treated with ipi + nivo fulfilled criteria for CNS irAE (Table 1 and case descriptions in Data Supplement). The patients had symptoms of CNS inflammation and improved after treatment with immunosuppression. Tumour progression or infection was ruled out. Lumbar puncture confirmed CNS inflammation by lymphocytosis (7/8) and/or an increased albumin quotient (6/8). In the two patients with a normal albumin quotient, lumbar puncture was done after administration of the first dose(s) of corticosteroids. Immune cell phenotyping was performed in 4 patients and the mean CD4/CD8 ratio in CSF was 3.25 ± 1.2 (standard deviation) (range 2.5–5). The CSF concentrations of axonal damage marker NfL was high (6/9), in particular in patients with severe neurological symptoms (Table 1). No neuronal autoantibodies were detected in blood (Supplmentary Table S3) or CSF (Supplmentary Table S4) of patients with CNS irAE.

**Table 1:** Patients with CNS irAE.

	Case 1^a^	Case 2	Case 3	Case 4	Case 5	Case 6	Case 7	Case 8	Case 9
Severity grade^b^	Fulminant	Fulminant	Severe	Severe	Severe	Moderate	Moderate	Moderate	Moderate
Type^b^	irDemyelinating	irEncephalitis	irEncephalitis	irMeningoencephalitis	irMeningoencephalitis	irEncephalitis	irMeningitis	irEncephalitis	irEncephalitis
Days since first ipi + nivo	72	108	152	25	22	36	34	17	69
Days in hospital	71	83	9	7 + 5	12	4	12	14	5
Comorbidities	Type 2 diabetes	Hypertension	None	None	Hypertension	None	None	Hypertension	Type 2 diabetes
Immune suppression^c^	CORT, CP, MMF, IVIG	CORT, MMF	CORT	CORT	CORT	None	CORT	CORT	CORT
Symptoms and signs	**Peak** Loss of consciousness Central respiratory depression Total paresis in lower body **Recovery** Walks with support	**Peak** Headache Disorientation Leg paraparesis Loss of reflexes in lower extremities **Recovery** Walks short distances. Disrupted balance	**Peak** Severely reduced leg strength Loss of sensation in legs Moderately disturbed balance **Recovery** Lightly disturbed balance	**Peak** Headache Fever Photophobia New onset spasm right leg **Recovery** Spasm right leg	**Peak** Headache Fever Fatigue Impaired motor function right leg Nausea Peripheral vision loss **Recovery** Full recovery	**Peak** Disorientation Disturbed balance Memory loss **Recovery** Fatigue Tardiness	**Peak** Headache Fever Photosensitivity Stiff neck Myalgia **Recovery** Mild headache	**Peak** Confusion Fever **Recovery** Full recovery	**Peak** Disorientation Confusion Unsteady gait **Recovery** Full recovery
Brain MRI—overall	Diffuse lesions in brain and spinal cord^a^	Normal	Normal	Normal	Normal	Normal	Normal	Normal	Normal
MRI—Response in brain metastases	Complete remission^a^	(No brain mets.)	(No brain mets.)	Partial remission	(No brain mets.)	(No brain mets.)	Mixed response	Stable	(No brain mets.)
CSF—brain damage markers	GFAp: 74 × ULN NFL: 94 × ULN Tau: 3.5 × ULN	GFAp: 28 × ULN NFL: 4 × ULN Tau: 3.5 × ULN	GFAp: 1.1 × ULN NFL: 2.9 × ULN Tau: 1.1 x ULN	GFAp: 1.3 × ULN NFL: 17.8 × ULN Tau: 1.7 x ULN	GFAp: Normal NfL: 5 x ULN Tau: Normal	GFAp: 1.2 × ULN NFL: 5.8 × ULN Tau: 1.1 × ULN	GFAp: Normal NFL: Normal Tau: Normal	GFAp: Normal NfL: Normal Tau: 1.5 × ULN	GFAp: Normal NfL: Normal Tau: Normal
CSF—inflammation	Protein: 6.9 × ULN Lymphocytes: 4 × ULN	Protein: 3.4 × ULN Lymphocytes: 10 × ULN	Protein: 1.3 × ULN Lymphocytes: Normal	Protein: 1.2 × ULN Lymphocytes: 4 x ULN	Protein: 1,7 × ULN Lymfocytes: 5.3 × ULN	Protein: 3.8 × ULN Lymphocytes: 6.25 x ULN	Protein: Normal Lymphocytes: 3.5 x ULN	Protein: 1.4 × ULN Lymfocytes: Not analysed	Protein: Normal Lymfocytes: 2.3 × ULN
CSF—immune cell phenotype	Not analysed	88% T-cells (CD4:CD8 5:2) 2% B-cells (non-clonal) 10% NK-cells	86% T-cells (CD4:CD8 3:1) 1% B-cells (non-clonal) 13% NK-cells	Not analysed	Not analysed	94% T-cells (Ratio CD4:CD8 5:1) <1% B-cells (non-clonal) 6% NK-cells	98% T-cells (CD4:CD8 5:2) <1% B-cells (non-clonal) 2% NK-cells	Not analysed	Not analysed
CSF—infection^d^	Virus: Neg Bacteria: Neg	Virus: Neg Bacteria: Neg	Virus: Neg Bacteria: Neg	Virus: Neg Bacteria: Neg	Virus: Neg Bacteria: Neg	Virus: Neg Bacteria: Neg	Virus: Neg Bacteria: Neg	Virus: Neg Bacteria: Neg	Virus: Neg Bacteria: Neg
CSF—autoantibodies^e^	Negative	Negative	Negative	Negative	Negative	Negative	Negative	Not analysed	Negative
Other concomittant irAE	Hepatitis grade 1	Hepatitis grade 2	Hepatitis grade 2	Hepatitis grade 1	Hepatitis grade 1	Hepatitis grade 2	Hepatitis grade 3	Hepatitis grade 1	Hepatitis grade 1 Arthralgia grade 2

MRI, magnetic resonance imaging; CSF, cerebrospinal fluid; ULN, upper limit of normal; GPAp, glial fibrillary acidic protein; NFL, neurofilament light; Tau, Tau protein; CXCL13, Chemokine (C-X-C motif) ligand 13; S100B, S100 calcium-binding protein B.

a Patient described in detail in PMID: 34215689.

b Severity grade and type of CNS irAE was determined using consensus criteria according to Guidon et al. PMID: 34281989.

c CORT, cortocosteroids; CP, cyclophosphamide; MMF, mycophenolate mofetil; IVIG, intravenous Immunoglobulin.

d Bacterial culture in CSF, Filmarray meningitis panel: Herpes simplex type 1 and 2, Varicella zooster virus, Enterovirus, Human herpes virus 6, Human parechovirus, Cytomegalovirus, Streptococcus pneumonia, Hemophilus.

e Detailed description in Supplmentary Table S4.

The patients ranged in age from the early 30s to the mid-70s, 59 ± 14.8 years (mean ± SD). All patients required hospitalization for a median 12 days (IQR: 9–14 days, range 2–83 days); two patients needed intensive care. Neurological symptoms occurred within a median 36 days (IQR: 25–72 days, range 17–152 days) after the first dose of ipi + nivo. After diagnosis of CNS irAE the patients were followed up for 458 ± 193 days (mean ± SD). Five patients recovered fully; the remaining four patients had a slower partial recovery. Magnetic resonance imaging of the brain showed no abnormalities in all patients except one, described in detail previously,^8^ who had near-fatal acute disseminated encephalomyelitis. No patient died from CNS irAE. Two patients died from tumour progression, 274 and 788 days after diagnosis of CNS irAE.

### Cerebrospinal fluid analysis in CNS irAE, herpes simplex virus encephalitis and controls

Patients with CNS irAE or herpes simplex virus encephalitis had significantly higher number of lymphocytes, higher albumin quotient and higher NfL in cerebrospinal fluid than SLE controls without CNS inflammation (Supplmentary Fig. S1). There were no significant differences between patients with CNS irAE and herpes simplex virus encephalitis. Collectively, the analyses indicate a similar inflammatory profile in CSF in patients with CNS irAE and patients with herpes simplex virus encephalitis. Cytokines and chemokines in cerebrospinal fluid were analysed in a subset (7/9) of patients with CNS irAE (Supplmentary Table S5).

### S100B and NfL concentrations in blood during CNS irAE

**Fig. 1:**
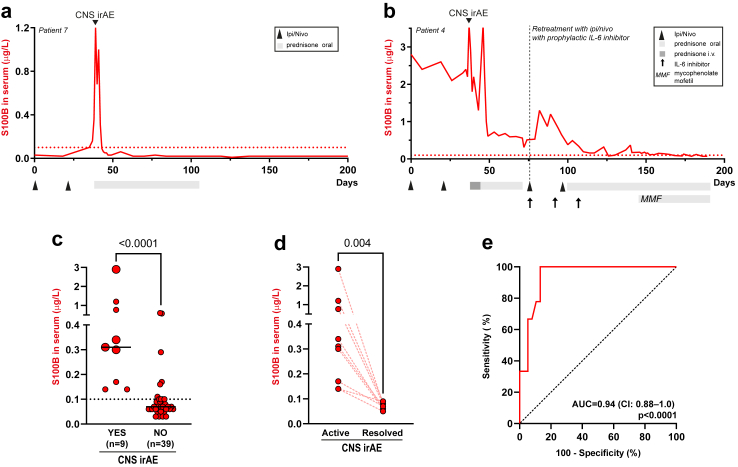
**S100B in serum in patients with or without CNS irAE.** (a) Serum S100B dynamics in patient with melanoma treated with ipilimumab (ipi) and nivolumab (nivo). The patient had extensive brain metastases and limited extracranial metastases (lymph nodes in abdomen) and was S100B negative. The patient developed neurological symptoms (photophobia, headache, fever, neck stiffness) after the second treatment. CNS inflammation was verified with lumbar puncture. There was a distinct peak in serum S100B at the time of CNS irAE which rapidly normalized when corticosteroid treatment was started. (b) Serum S100B concentrations in S100B positive patient with extensive brain metastases and extracranial metastases. The patient started ipi + nivo and responded remarkably well. As a sign of response, S100B in blood decreased. However, after two treatments he experienced neurological symptoms (fever, headache, photophobia, paresis) despite regression of brain metastases (and no oedema). Lumbar puncture verified CNS inflammation and he started iv methylprednisolone and improved. Notably, there was a distinct peak in S100B in blood at the time of CNS irAE. A second S100B peak was seen when iv methylprednisolone was changed to a lower dose oral prednisone. He was later re-treated with ipi + nivo with prophylactic IL-6 inhibition (sarilumab 200 mg subcutaneously every 2 weeks) and CNS irAE did not re-occur. (c) Peak serum S100B concentrations in ipi + nivo treated patients with (n = 9) or without (n = 39) CNS irAE. Large dots signify severe symptoms of CNS irAE; small dots signify moderate symptoms. Statistical significance was determined with the Mann–Whitney U test. Horizontal lines indicate median values. (d) Serum S100B concentrations in patients during active and resolved CNS irAE. The p-value refers to two-tailed sign test. (e) Receiver operating characteristic (ROC) curves of serum S100B to identify patients with CNS irAE. AUC, area under the ROC curve. CI, 95% confidence interval for AUC. The p values refer to the null hypothesis that the area under the ROC curve is 0.5. Dotted line in (a), (b) and (c) indicates upper limit of normal for S100B (0.1 μg/L).

**Fig. 2:**
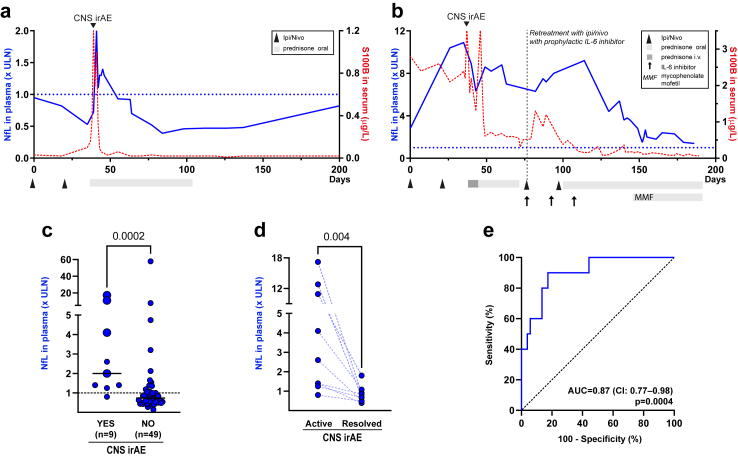
**Plasma NfL in patients with or without CNS irAE.** (a) Plasma NfL dynamics in the same patient as shown in Fig. 2a. Note that NfL (blue line, left y-axis) increases in blood as the patient developed CNS irAE. NfL peaked later and normalized more gradually than S100B (dotted red line; right y-axis). At baseline (before ipi + nivo) the patient had normal NfL concentration in plasma despite extensive brain metastases. (b) Plasma NfL dynamics in the patient depicted in Fig. 2b. NfL increased as the patient developed CNS irAE and slowly normalized after immunosuppressive treatment. The patient had increased NfL at baseline (before ipi + nivo), approximately three-fold the upper limit normal (ULN). At CNS irAE, plasma NfL increased to 12-fold the ULN and slowly normalized with immunosuppression. (c) Peak plasma NfL concentrations in ipi + nivo treated patients with (n = 9) or without (n = 49) CNS irAE. Plasma NfL was higher in patients with severe symptoms (large dots) than in patients with moderate symptoms (small dots). Statistical significance was determined with the Mann–Whitney U test. Horizontal lines indicate median values. (d) Plasma NfL concentration in patients during active and resolved CNS irAE. The p-value refers to two-tailed sign test. (e) Receiver operating characteristic (ROC) curves of NfL in plasma to identify patients with CNS irAE. AUC, area under the ROC curve. CI, 95% confidence interval for AUC. The p values refer to the null hypothesis that the area under the ROC curve is 0.5. Dotted line in (a), (b) and (c) indicates age-adjusted upper limit of normal for NfL (age less than 51 years <10 ng/L; 51–61 years <15 ng/L; 61–69 years <20 ng/L; >70 years <35 ng/L).

**Fig. 3:**
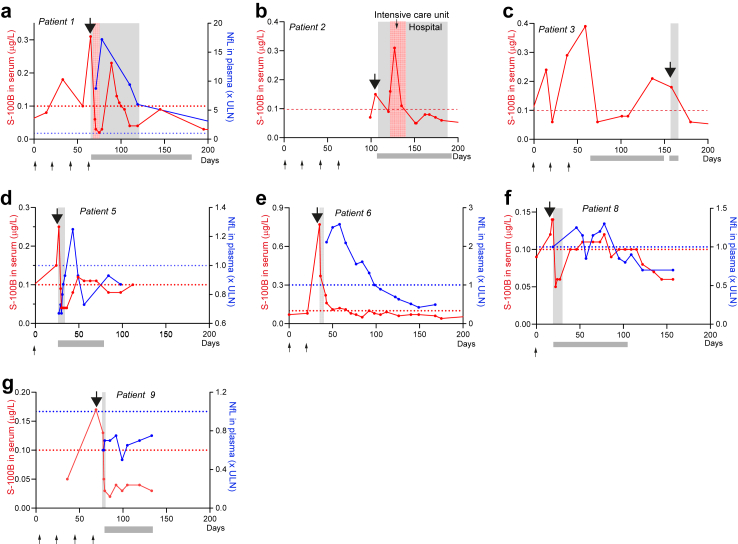
**Serum S100B and plasma NfL dynamics in patients with CNS irAE.** (a–g) Serum S100B (n = 7; red curves, left y-axis) and plasma NfL dynamics (n = 5; blue curves, right y-axis) in patients with CNS irAE. The arrows above the curves indicate time for first neurological symptom(s). The small arrows below the x-axis indicate the time for ipi + nivo treatment. The grey box under x-axis indicate period with immunosuppressive treatment. Patient numbers refer to Table 1 and to patient details included in Data Supplement. Dashed red (S100B, 0.1 μg/L) and blue (NfL, age-adjusted) lines indicate upper limit of normal. The age-adjusted upper limits of normal for NfL are: age less than 51 years <10 ng/L; 51–61 years <15 ng/L; 61–69 years <20 ng/L; >70 years <35 ng/L.

The dynamics in S100B and NfL in patients who developed CNS irAE are shown in Figs. 1, 2, and 3. Serum S100B increased in patients who developed CNS irAE and normalized rapidly after immunosuppression, often before clinical recovery (Fig. 1a and b, Fig. 3). In S100B negative CNS irAE patients, this produced a distinct peak in the S100B curve (Figs. 1a and 3). One patient with CNS irAE was S100B positive (Fig. 1b) and had S100B producing melanoma metastases in brain, muscle, subcutaneously, liver and lungs. As a consequence, serum S100B was very high before treatment start (almost 30 times above the normal upper limit) and decreased rapidly when metastases regressed. However, there were distinct peaks in serum S100B also in this patient when he experienced neurological symptoms due to CNS irAE (Fig. 1b). S100B peaks were rare in S100B negative patients with cancer without CNS irAE (Supplmentary Fig. S2a). 34 out of 39 of these patients had no S100B peaks (Supplmentary Fig. S2b). In the 5 patients with S100B peaks, 3 had no signs of irAE (Supplmentary Fig. S2c and f), 1 had hepatitis (Supplmentary Fig. S2d) and 1 colitis (Supplmentary Fig. S2e). Peak serum S100B concentration was higher in patients with CNS irAE than patients without CNS irAE (Fig. 1c) and normalized after immunosuppression (Fig. 1d). Peak serum concentration of S100B did not correlate with severity of CNS symptoms (Fig. 1c). The ROC curve showed a sensitivity of increased S100B for CNS irAE of 100% (95% confidence interval, CI, 70–100%) and a specificity of 79% (95% CI: 64–88%). The AUC was 0.94 (95% CI 0.88–1.0) (Fig. 1e).

Similar to S100B, NfL concentration in plasma increased in patients with CNS irAE. However, in most patients, NfL concentration increased, and peaked, later (median 11 days after the first neurological symptoms; range 0–62 days) (Figs. 2a and 3). In contrast to the rapid normalization of S100B, plasma concentrations of NfL decreased gradually after immunosuppression. NfL dynamics over time in two patients (same patients as in Fig. 1a and b) is shown in Fig. 2a and b, and for five patients in Fig. 3. In the remaining two patients, NfL was analysed twice, during active and resolved CNS irAE (included in Fig. 2c and d). NfL was higher in patients with CNS irAE than in patients with cancer without CNS irAE (Fig. 2c) and normalized after immunosuppression (Fig. 2d). Plasma concentration of NfL correlated to severity of CNS symptoms (Fig. 2c). The ROC curve showed a sensitivity of 89% (95% CI 57–99%) and a specificity of 74% (95% CI 60–84%) of increased plasma NfL for CNS irAE. The AUC was 0.87 (95% CI 77–98%) (Fig. 2e). The concentration of NfL in plasma correlated with the concentration in CSF (Spearman correlation test, r = 0.99, p = 0.006).

### CRP and ALT during CNS irAE

**Fig. 4:**
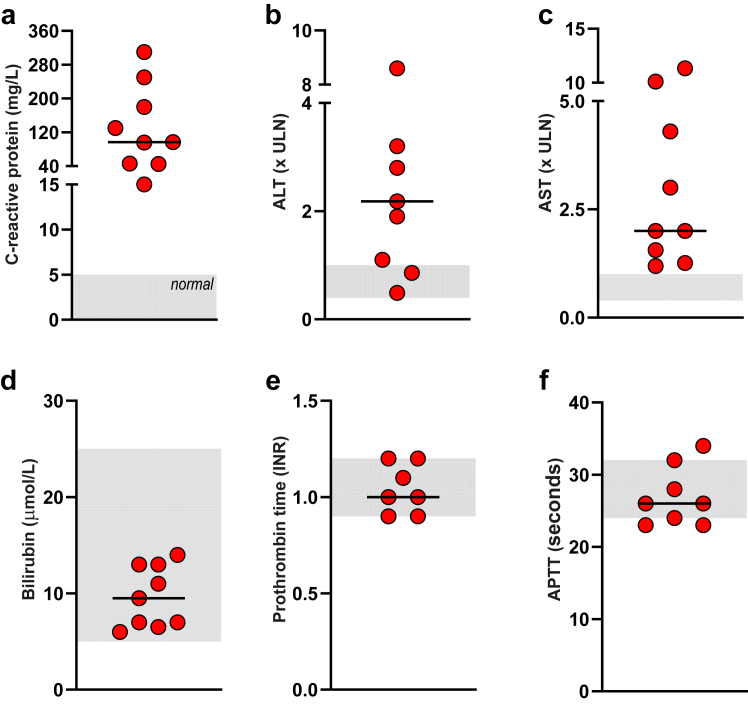
**Increased CRP, AST, and ALT during CNS irAE.** (a) Levels of CRP in blood were increased at the time of CNS irAE. (b and c) Concentrations of alanine aminotransferase (ALT) and aspartate aminotransferase (AST) in blood was also elevated at CNS irAE. (d, e, and f) Bilirubin, prothrombin time and activated partial thromboplastin time were within the normal range, indicating adequate liver function. Grey zones indicate normal ranges (a–f). Horizontal lines indicate median values (a–f).

**Fig. 5:**
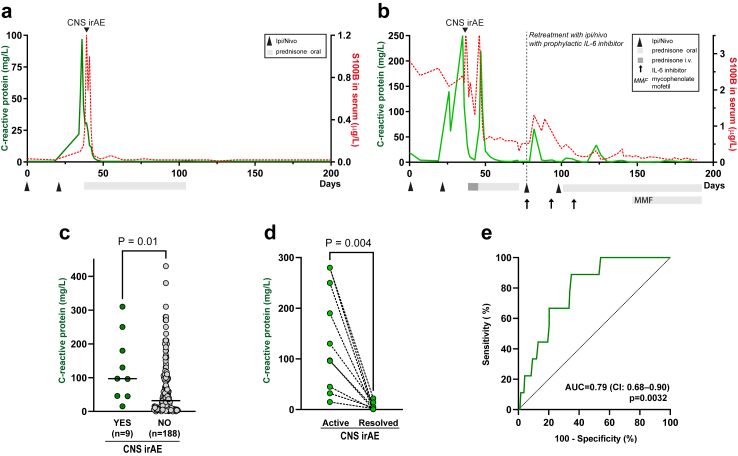
**CRP is increased during CNS irAE and covaries with S100B.** (a) CRP in blood (green line, left y-axis) and serum S100B (red dotted line, right y-axis) dynamics in the same patient as shown in Figs. 1a and 2a. CRP covaries with S100B and peaks during CNS irAE. CRP rapidly normalizes when immunosuppression is initiated. (b) CRP dynamics in the patient depicted in Figs. 1b and 2b. CRP increased after the second ipi + nivo treatment and peaked during CNS irAE. There is a second peak in CRP and S100B when intravenous methylprednisolone is switched to lower dose oral prednisone. When the patient was retreated with ipi + nivo with prophylactic IL-6 inhibitor, there was only a minor CRP peak, and the patient had no symptoms of CNS irAE. CRP and S100B concentrations covaried during both initial treatment and retreatment with ipi + nivo. (c) Peak CRP concentrations in ipi + nivo treated patients with (n = 9) or without (n = 188) CNS irAE. Statistical significance was determined with the Mann–Whitney U test. Horizontal lines indicate median values (d) CRP concentration in patients during active and resolved CNS irAE. Th p-value refers to two-tailed sign test. (e) Receiver operating characteristic (ROC) curves of CRP in identify patients with CNS irAE. AUC, area under the ROC curve. CI, 95% confidence interval for AUC. The p values refer to the null hypothesis that the area under the ROC curve is 0.5.

**Fig. 6:**
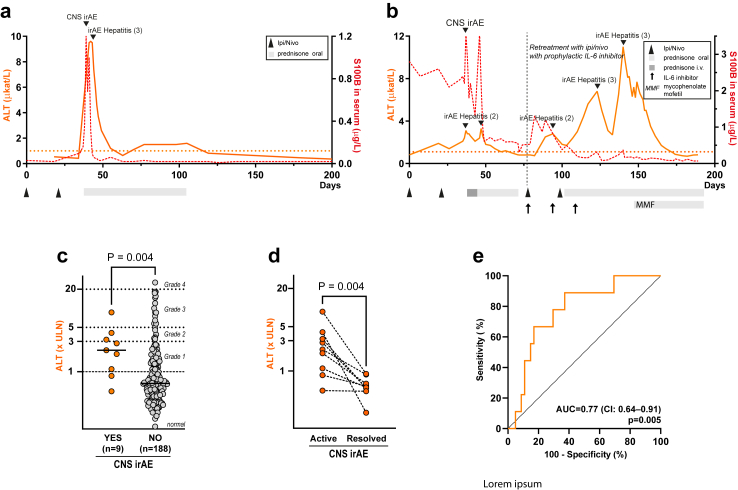
**ALT is increased during CNS irAE and covaries with S100B.** (a) ALT in blood (orange line, left y-axis) and serum S100B (red dotted line, right y-axis) dynamics in the same patient as shown in Figs. 1a and 2a. ALT peaked (grade 3 hepatitis) during CNS irAE and normalized when immunosuppression was initiated. ALT concentrations covaried with both S100B and CRP (Fig. 5). (b) Serum ALT dynamics in the patient depicted in Figs. 1b and 2b. ALT concentrations increased moderately (grade 2 hepatitis) after the second ipi + nivo treatment and peaked during CNS irAE and again when intravenous methylprednisolone was switched to oral prednisone. When retreated with ipi + nivo with prophylactic IL-6 inhibitor, the patient developed a more severe hepatitis (grade 3) which initially responded to corticosteroids but later required addition of mycophenolate mofentil. (c) Peak ALT concentrations in ipi + nivo treated patients with (n = 9) or without (n = 188) CNS irAE. Statistical significance was determined with the Mann–Whitney U test. Horizontal lines indicate median values. (d) ALT concentration in patients during active and resolved CNS irAE. The p-value refers to two-tailed sign test. (e) Receiver operating characteristic (ROC) curves of ALT in blood to identify patients with CNS irAE. AUC, area under the ROC curve. CI, 95% confidence interval for AUC. The p values refer to the null hypothesis that the area under the ROC curve is 0.5. Dotted line in (a) and (b) indicates sex-adjusted upper limit of normal for ALT (men, 1.1 μkat/L; women, 0.75 μkat/L). Dotted lines in (c) indicates cut-off for grade 1–4 irAE hepatitis.

Patients with CNS irAE had a simultaneous increase in CRP level above normal (9/9 patients) and most CNS irAE patients had an increase in AST and/or ALT above normal (8/9 patients) (Fig. 4a–c). Bilirubin, prothrombin time and activated partial thromboplastin time in blood were normal indicating adequate liver function (Fig. 4d–f). Other routine blood tests, including creatinine and creatinine kinase, were normal or low suggesting adequate kidney function and low muscle mass (Supplmentary Fig. S3). The dynamics in CRP (Fig. 5a and b and Supplmentary Fig. S4) and ALT/AST (Fig. 6a and b) in blood over time co-varied with neurological symptoms and serum concentration of S100B. The peak CRP and ALT levels in CNS irAE patients (n = 9) was higher than the peak CRP and ALT values (within 200 days from the first treatment) in the identically treated patients without CNS irAE (n = 188) (Figs. 5c and 6c and Supplmentary Fig. S4). CRP and ALT concentrations normalized after immunosuppression (Figs. 5d and 6d). The sensitivity of an increased concentration of CRP for CNS irAE was 100% (95% CI 70–100%) and specificity was 27% (95% CI 21–34%). The AUC was 0.79 (95% CI 0.68–0.90) (Fig. 5e). For ALT the sensitivity was 78% (95% CI 45–96%) and the specificity was 67% (95% CI 60–74%). The AUC was 0.77 (95% CI 0.64–0.91) (Fig. 6e).

### Increase in plasma S100B, NfL, ALT, and CRP after ipi + nivo in patients without CNS irAE diagnosis

Analysis of frozen plasma samples revealed rapid increase in plasma NfL during ipi + nivo treatment in one patient with melanoma in the control group without CNS irAE (Supplmentary Fig. S5a and b). In addition, S100B, CRP, AST also increased after ipi + nivo and peaked when the patient was hospitalized due to grade 3 hepatitis (Supplmentary Fig. S5c and d). At this time the patients suffered from acute fatigue and fever, symptoms that are usually not associated with irAE hepatitis. The patient who was around 50 years old was healthy, with no history of stroke or neurological disease, no cardiovascular diseases, no medications, and no brain metastases. There was no sign of peripheral nerve dysfunction or liver failure (normal APTT, normal prothrombin time (INR), normal platelet count). Creatinine and creatinine kinase were also normal. Upon intravenous treatment with high-dose corticosteroids, the concentrations of S100B, CRP, and ALT in blood normalized, and the patients’ condition temporarily improved. Corticosteroids were gradually reduced but the patient rapidly deteriorated and unexpectedly died. No autopsy was performed. The last NfL analysed was very high, almost 60 times the upper normal limit.

### S100B and NfL in blood in patients with or without brain metastases

We also investigated if there was a difference in concentration of S100B or NFL in blood in patients with or without brain metastases. In patients with CNS irAE, three of nine had brain metastases (Table 1). S100B (3/3) and NfL concentrations (2/3) in blood were analysed in these patients before ipi + nivo treatment. In patients without CNS irAE, seven of 39 (S100B, Supplmentary Table S1) and six of 49 (NfL, Supplmentary Table 2) had brain metastases. There were no significant differences in S100B or NfL concentrations in patients with versus without brain metastases (Supplmentary Fig. S6).

## Discussion

This study shows that analysis of the brain damage markers S100B and NfL in blood facilitates early diagnosis and clinical monitoring of CNS irAE. CNS irAE were also associated with high CRP levels and irAE hepatitis (increased ALT and/or ALT). Finally, the incidence of CNS irAE in our cohort of patients treated with ipi + nivo is higher than previously reported.

Serum S100B concentrations increased early in patients with CNS irAE, in some cases before neurological symptoms were apparent, possibly indicating blood–brain barrier dysfunction.^13^^,^^29^, ^30^, ^31^ S100B leaks from CSF to blood through a dysfunctional blood–brain barrier and increases the serum concentration of S100B (serum S100B is normally <10% of the CSF concentration). Indeed, most of the patients with CNS irAE had signs of blood brain–barrier dysfunction as indicated by an increased albumin quotient. Serum S100B normalized quickly (within hours) after corticosteroids were administered, perhaps because the treatment decreased blood brain–barrier permeability and therefore reduced leakage of S100B into serum.^32^ S100B has a short serum half-life (minutes to hours) and therefore normalizes quickly when leakage into the blood decreases.^30^ These observations suggest that increased permeability of the blood–brain barrier is an early event in CNS irAE and can be detected by analysis of serum S100B. Increased blood–brain barrier permeability may also facilitate immune cell infiltration into the CNS and increase the severity of CNS irAE.

Both plasma and CSF concentrations of NfL correlated with the severity of neurological symptoms during CNS irAE. Plasma and CSF NfL concentrations also correlate with disease severity in other neuroinflammatory disorders, such as multiple sclerosis and autoimmune encephalitis.^15^, ^16^, ^17^, ^18^ In contrast to S100B, plasma NfL decreased slowly and in parallel with the improvement of neurological symptoms. In contrast to S100B, NfL has a long plasma half-time of weeks to months.^33^ Therefore, NfL was superior to S100B for assessing disease severity and monitoring clinical recovery. S100B, on the other hand, was a more sensitive marker and, in most patients, indicated CNS irAE earlier than NfL.

Checkpoint inhibitor-induced autoimmune irAE can affect any organ of the body in an unpredictable way.^5^^,^^6^ Well-established and validated blood tests such as AST and ALT (liver), creatinine (kidney), thyroxin and thyroid stimulating hormone (thyroid gland), and troponin (heart) are currently used tools to diagnose and monitor organ-specific irAE.^34^ Our finding that established brain damage markers S100B and NfL are useful in diagnosis of CNS irAE is therefore not surprising. The fact that both S100B and NfL are clinically validated and broadly explored markers is a strength. However, and in similarity to other clinically used irAE markers, neither S100B nor NfL are specific. NfL is also expressed by peripheral nerves and S100B is expressed in several extracerebral cell types.^35^ Therefore, it is important to exclude causes other than irAE in each clinical situation. Serum cytokine profiles,^36^ T cell receptor clonality,^37^ T cell phenotype,^8^^,^^38^ genetic factors^39^ and B cell phenotype^39^^,^^40^ are also explored as irAE markers. Importantly, some of these markers may indicate risk of irAE early during treatment or even before treatment.

Patients with CNS irAE also had increased concentration of CRP in blood which normalized during the patients’ recovery. Elevated concentration of CRP is a feature of several other irAE as well as infection, surgery, and, sometimes, tumour progression.^41^^,^^42^ Therefore, the specificity of CRP to predict CNS irAE is low. However, a high CRP in combination with high S100B and/or NfL indicates CNS irAE. CRP is produced by hepatocytes in response to high concentrations of inflammatory cytokine IL-6 in blood.^41^^,^^43^ IL-6 inhibition has also shown promising effect in management of irAE^44^^,^^45^ and there are ongoing trials to investigate if IL-6 inhibition can prevent irAE in patients treated with ipi + nivo (NCT04940299, NCT03999749). In this report, CNS irAE did not recur in the one patient who received prophylactic IL-6 inhibition before re-treatment with ipi + nivo after recovering from a previous episode of CNS irAE. To conclude, our observations suggest that IL-6 may contribute to development of CNS irAE, and it is possible that the risk of CNS irAE is reduced by prophylactic IL-6 inhibition.

Nine out of nine patients with CNS irAE in this study also had irAE hepatitis as defined by increased AST and/or ALT. In identically treated patients with cancer without CNS irAE 26% developed irAE hepatitis, a similar frequency as previously reported.^46^ Therefore, an isolated increase in AST or ALT is unlikely to indicate CNS irAE. However, the observation of concurrent CNS and hepatitis irAE is interesting and indicates that there may be shared underlying immune mechanisms between these two adverse events. If these immune mechanisms are defined, it would open up for more specific interventions than currently used broad immunosuppressive treatment with high dose corticosteroids. An alternative, but less likely, explanation to concurrent CNS symptoms and increased AST/ALT could be that acute liver dysfunction, secondary to irAE hepatitis, induces liver encephalopathy. Liver encephalopathy is defined as neurological symptoms due to accumulation of ammonium and other CNS toxic metabolites in patients with severe liver failure.^47^ However, the patients in our cohort had mild to moderate hepatitis and normal liver function tests as well as normal bilirubin in blood. Furthermore, there was no correlation between the degree of irAE hepatitis and the severity of CNS symptoms. Finally, all patients had evidence of inflammation in cerebrospinal fluid which is not typical of liver encephalopathy. Collectively, liver dysfunction is a less likely cause of CNS symptoms in our cohort of patients.

Interestingly, in one patient without diagnosis of CNS irAE, ipi + nivo treatment was followed by rapidly increased concentrations of NfL, S100B, CRP and ALT in blood. Consequently, this patient had the same pattern in blood as patients with CNS irAE. The NfL analysis was performed on biobanked samples after death. The rapid increase in NfL following ipi + nivo was at a magnitude (almost 60 times the upper limit of normal) that is seen after severe traumatic injury to the head.^48^ This was unexpected because except for the melanoma (without brain metastases), the patient was healthy (no other medications, no comorbidities), no signs of new CNS pathologies (MRI of the brain was normal) and no symptoms of peripheral nerve damage. One possibility is that the patient may have had an undiagnosed CNS irAE based on symptoms and the laboratory abnormalities. In agreement with this hypothesis, S100B, CRP, and ALT normalized when the patient was treated with immunosuppression.

In addition to pleocytosis and/or elevated protein, cerebrospinal fluid from patients with CNS irAE had high concentrations of IL-8, CXCL10 and CXCL13. In contrast, IL1β, IL-2, IL-4, IL-5, IL-6, IL-10, IL-12, IFNγ, and TNF-α concentrations were normal in most patients. CSF concentrations of IL-8, CXCL10 and CXCL13 are frequently elevated in multiple sclerosis and other neuroinflammatory diseases.^49^, ^50^, ^51^ However, the exact role of these proteins in neuroinflammation is not known. No neuronal autoantibodies were detected in serum or CSF in our cohort of CNS irAE patients. In other cohorts, neuronal autoantibodies have been reported in a frequency varying from 30 to 50% of CNS irAE patients.^9^^,^^52^

CNS irAE was diagnosed in nearly 5% of our patients—a higher incidence than previously reported. In a systematic review of all major clinical trials of checkpoint inhibitors, the frequency of severe neurological irAE was below 1%, varying between 0.4% for PD1 inhibitors (nivo/pembro) and 0.7% for the combination of PD1 and CTLA4 inhibitors (ipi + nivo).^12^ In better agreement with our data, a recent real-world data review suggested a higher frequency,^7^ finding severe neurological irAE (grade 3–4) in 2.8% of patients treated with both PD1 and CTLA4 inhibitors and in 1% of those treated with PD1/PDL1 inhibitors. The discrepancy between studies may reflect the difficulty of diagnosing CNS irAE, especially early in the course of the disease. Early diagnosis is important because patients may develop severe complications, or even die, without immunosuppressive treatment.^8^^,^^9^ In addition to the suffering of patients, there is a high cost for the health care system. The nine patients with CNS irAE in our cohort spent a mean time of 24 days in the hospital. Patients who were diagnosed late had the longest hospital stays and experienced prolonged neurological complications, whereas those who were diagnosed early and rapidly received immunosuppression recovered quickly. Monitoring the S100B and NfL concentrations in ICI-treated patients could facilitate the early diagnosis and treatment of CNS irAE.

Our study had several limitations. First, since it was a retrospective single-centre study the results might not be representative of a broader patient population. In addition, methodological limitations include uncontrolled confounding in between-group comparisons and regression to the mean in within group comparisons. Therefore, it is important to verify the findings in controlled prospective studies. Another limitation was the selection of controls for NfL. NfL is not routinely analysed, and biobanked plasma samples from patients with cancer without CNS irAE were used as controls for NfL. Most of NfL patients with cancer without CNS irAE were treated with single nivo or pembro and not ipi + nivo. One major strength is that S100B was measured at least monthly in all patients with melanoma, as it is both a melanoma marker and a brain damage marker. As a result, repeated serum S100B analyses were available from ICI-treated S100B negative patients with melanoma with and without CNS irAE. This analysis showed that distinct peaks in S100B were detected in patients with CNS irAE but rarely in patients without CNS irAE. As shown in this study, CNS irAE are best detected and monitored by following the variations in S100B, NfL and CRP in blood over time.

Data presented here illustrate that the analysis of S100B and NfL in blood over time is a powerful, accessible, and simple clinical tool to detect and monitor CNS irAE. Patients with increased concentration(s) of S100B and NfL in blood should undergo neurological examination, brain MRI and lumbar puncture. Simultaneous increase in CRP and AST/ALT may indicate a higher risk of CNS irAE. The results presented here need to be confirmed in prospective studies.

## Contributors

ML and SB conceived and designed the study. ML, SB, AP, ZZ, HAR, JS, HZ, MS, A-C L, AR, and LN acquired data. All authors helped analyse and interpret the data. SB and ML did the literature search and wrote the manuscript. ML and SB have directly assessed and verified the underlying data reported in the manuscript. All authors helped review and/or revise the manuscript. All authors have read and approved the final version of the manuscript. SB and ML are responsible for the decision to submit the manuscript.

## Data sharing statement

Anonymized data will be made available upon requests directed to the corresponding author. Proposals will be reviewed and approved by the authors based on scientific merit.

## Declaration of interests

SB has received lecturing fees from Bristol Myers Squibb. ML has received lecturing fees from Bristol Myers Squibb, MSD, Roche, and Novartis. LN has received lecturing fees from Bristol Myers Squibb, Glaxo Smith Kline, MSD, Novartis, and Pierre Fabre. LN has received advisory board fees from Bristol Myers Squibb, Glaxo Smith Kline, MSD, Novartis, Pierre Fabre and Zealth. LN has stocks/ownership in SATMEG Ventures. HZ has received consulting or advisory board fees from Abbvie, Acumen, Alector, Alzinova, ALZPath, Annexon, Apellis, Artery Therapeutics, AZTherapies, CogRx, Denali, Eisai, Nervgen, Novo Nordisk, Optoceutics, Passage Bio, Pinteon Therapeutics, Prothena, Red Abbey Labs, reMYND, Roche, Samumed, Siemens Healthineers, Triplet Therapeutics, and Wave. HZ has received lecturing fees from Fujirebio, Alzecure, Cellectricon, Biogen and Roche. HZ is a co-founder of Brain Biomarker Solutions in Gothenburg AB, which is a part of the GU Ventures Incubator Program. The other authors report no disclosures.

## Appendix ASupplementary data

Supplementary Result, Figs. S1–S6, and Tables S1–S5
