# Supplementary material for: Concentrations of S100B and neurofilament light chain in blood as biomarkers for checkpoint inhibitor–induced CNS inflammation
Source: eBioMedicine. 2024 Jan 3;100:104955. doi: 10.1016/j.ebiom.2023.104955 (PMC10796943; doi:10.1016/j.ebiom.2023.104955)
Supplement: Supplementary Result, Figs. S1–S6, and Tables S1–S5 [file mmc1.docx]

**DATA SUPPLEMENT**

**CONTENTS**

***SUPPLEMENTARY RESULTS.............................................................................................................. 2***

**CNS irAE patient cases ….………………………………………………………………………………………….………..…….…...2**

***SUPPLEMENTARY FIGURES.............................................................................................................. 7***

**Figure S1. Lymphocytes, albumin quotient and NfL in cerebrospinal fluid from CNS irAE patients, herpes simplex virus encephalitis patients and SLE controls…………………………….…….…………..…….…...7**

**Figure S2. Serum S100B dynamics in ipi+nivo treated patients without CNS irAE…….…………..…….…...8**

**Figure S3.** **Creatinine and creatinine kinase in blood in patients with CNS irAE……..…….…………………9**

**Figure S4.** **Covariation of S100B and CRP in patients with CNS irAE ………………..……..…………………….10**

**Figure S5. Patient with rapid increase in NfL following ipi + nivo treatment…...................................11**

**Figure S6. Concentrations of S100B and NfL in blood in patients with or without brain metastases...12**

***SUPPLEMENTARY TABLES ..............................................................................................................13***

**Table S1. S100B cohort – Ipi+nivo treated patients without CNS irAE …………………………….................13**

**Table S2. NfL cohort – Ipi+nivo or nivo/pembro treated patients without CNS irAE .........................14**

**Table S3. Neuronal autoantibodies in blood ………………………………………..……………...……….…..….........15**

**Table S4. Neuronal autoantibodies in CSF……………………………………………..……………………………...........16**

**Table S5. Cytokines and chemokines in CSF……………………………………………..…………………………...........17**

**SUPPLEMENTARY RESULTS**

**CNS irAE patient cases**

***Patient 1*** *–* Patient described in detail in previously (PMID: 34215689). S100B and NfL dynamics is shown in Fig. 3a.

***Patient 2*** *–* A patient in their 70’s with advanced renal clear cell carcinoma with metastases to both adrenal glands was started on first-line treatment with ipilimumab 1 mg/kg plus nivolumab 3 mg/kg every 3 weeks. The patient felt well until 6 weeks after the fourth treatment when the patient experienced fatigue, vertigo, confusion, and discrete pyrexia. At that time S100B had increased to 0⋅15 μg/L (reference <0⋅10 μg/L). The patient’s condition rapidly declined over the following two days when they became disoriented and developed unsteadiness needing assistance just to walk a few meters. The patient had lost control of their bladder function and needed a urine catheter. The patient was admitted to hospital where CT-scans of body and brain was normal. CSF showed increased in lymphocytes, protein, CXCL13, and brain damage markers (NfL, GFAP and Tau) were elevated. EEG showed an encephalopathic pattern. Prednisolone 0⋅5 mg/kg was started but the patient’s condition deteriorated further over the following week, becoming paraplegic and a lowered consciousness. At this point, S100B peaked at 0⋅31 μg/L. The patient was put on high dose methylprednisolone 1000 mg intravenously daily and mycophenolate mofetil 1000 mg twice daily. S100B normalized after immunosuppression. The patient’s recovery was hampered by contracting a severe covid-infection after being infected by a fellow patient during his hospital stay. 9 months later the patient was free of immunosuppressive treatment and had a stable cancer response. The patient was then able to walk with assistive devises. Plasma NfL was analysed twice, it was high during intensive care (12⋅8 times upper limit of normal) and normalized ten months later (0⋅7 times upper limit of normal) when the patient had made significant, but not full, recovery.

***Patient 3*** *–*  A patient in their 60s with clear cell kidney cancer metastasized to the liver received third-line treatment with ipilimumab 1 mg/kg plus nivolumab 3 mg/kg after progressing on both kabozantinib and nivolumab single therapy. After the third treatment the patient developed a grade 3 immune related hepatitis and at this point there was a simultaneous peak in S100B (0⋅39 μg/L). The patient started prednisolone 1 mg/kg, and AST, ALT and 100B normalized. Treatment evaluation showed tumour progression and the treatment was changed to sunitinib. Prednisolone was slowly tapered but had to be increased again when patient had a new flair of hepatitis and a new S100B peak (0⋅21 μg/L). Approximately 3 weeks after the second flair of hepatitis (during tapering of prednisolone) the patient suddenly developed a mild paraparesis in the lower extremities, more pronounced on the right side and needed assistive devices to walk. Serum S100B was still increased (0⋅18 μg/L). The patient was admitted to a neurology ward. The spinal fluid showed no signs of infection but increased protein, increased CXCL13 and elevated brain damage markers (GFAP, NfL and Tau). MRI of the brain and spinal cord was normal. Both sensory and motoric neurography was normal. Electromyography showed decreased muscle activation. The combined clinical assessment by the team of neurologists was a central inflammatory disorder rather than Guillain-Barré syndrome (which was the initial suspicion). High dose methylprednisolone (1000 mg intravenously daily) was given for 5 days, and the patient experienced a rapid recovery the patient was able to walk without assistance within a month. At this point, S100B was normalized. The full S100B dynamics is shown in figure 3c. Plasma NfL was analysed twice, during the hospital stay (1⋅4 times the upper limit of normal) and four months later (0⋅4 times the upper limit of normal).

***Patient 4*** *–*  A patient in their early forties with melanoma with metastases in brain, liver, lungs, and bone, started treatment with ipilimumab 3 mg/kg plus nivolumab 1 mg/kg. Two weeks after the second treatment, the patient was admitted to the Oncology ward because of severe headache, fever, spasms in the right leg as well as photosensitivity. Lymphocytes, CXCL10, CXCL13 and brain damage markers (GFAP, NfL and Tau) were elevated in cerebrospinal fluid but screening for bacteria and viruses was negative. Brain MRI showed partial regression of several metastases. Simultaneously the patient had grade two hepatitis. The patient was put on high dose cortisone (methylprednisolone 1 mg/kg intravenously) and drastically improved within 24 hours. The cortisone treatment was slowly tapered over the course of 5 weeks, the patient felt well, and the liver enzymes were normalized but brain MRI showed new metastasis. The patient received a third treatment dose of ipilimumab plus nivolumab with addition of IL-6 inhibitor (Sarilumab 200 mg sc.) with the intention to prevent recurrence of severe neurological irAE or hepatitis. This time the patient tolerated the treatment well and a fourth treatment with addition of Sarilumab was given. After the fourth dose there were no signs of recurring neurological toxicity however, the patient developed a grade 3 hepatitis, improving on iv methylprednisolone and mycophenolate mofetil. Radiological evaluation three weeks after the fourth treatment showed a significant decrease in size and activity of brain metastasis as well as a partial response of extra cranial metastases. S100B and NfL dynamics for the patient is shown in Fig. 1b and Fig. 2b.

***Patient 5*** *–* A patient in their fifties with uveal melanoma metastasized to the liver started treatment with ipilimumab 3 mg/kg plus nivolumab 1 mg/kg. 19 days after the first treatment the patient developed a headache and fever that worsened during the next couple of days as well as developing severe fatigue, nausea, double vision, and impaired muscle function of the right leg. The patient was admitted to the Oncology ward. MRI of brain and spine were normal. Analysis of cerebrospinal fluid confirmed a CNS inflammation with lymphocytosis, increased CXCL13 and increased NfL. Simultaneously the patient had a grade one hepatitis. The patient started treatment with high dose methylprednisolone 1000 mg daily for three days followed by methylprednisolone 2 mg/kg. The symptoms gradually improved over the following 10 days. The dose of corticosteroids was tapered slowly. S100B and NfL dynamics is shown in Fig 3d. Note that S100B peaks at the same time as neurological symptoms whereas NfL peaks later.

***Patient 6*** *–* A patient in their seventies with multiple melanoma lung metastases started treatment with nivolumab (480 mg every month) in March 2021. In September 2021, after 6 months of treatment, evaluating CT-scan showed progression of lung metastases and the treatment was escalated to ipilimumab 3 mg/kg plus nivolumab 1 mg/kg every three weeks. Two weeks after the second ipi + nivo the patient turned up without an appointment, confused and disoriented at the Oncology Department. The patient had a moderately affected balance and a short-term memory loss. The patient was admitted to the hospital for investigation and serum S100B was increased to 0⋅8 μg/L. CT scan of the brain was normal. Analysis of cerebrospinal fluid showed lymphocytosis, high concentration of CXCL10 and CXCL13 and elevated brain damage markers (GFAP, NfL and Tau). CT scan of the chest showed regression of lung metastasis. Simultaneously, the patient had elevated liver enzymes, interpreted as immunotherapy-induced grade 2 hepatitis. Within the next couple of days, the patient recovered gradually from acute confusion and hepatitis without any immunosuppressing therapy being administered. S100B return to levels below the upper limit of normal. The S100B and NfL dynamics are shown in Fig. 3e. Note that S100B peaks and normalizes before NfL.

***Patient 7*** *–* A patient in their late twenties was diagnosed with metastatic melanoma including multiple symptomatic brain metastases. The patient was started on BRAF and MEK-inhibitor as well as high dose betamethasone treatment. The patient had a good partial response of the brain metastases and, after 9 weeks, when the betamethasone treatment had been tapered down to 1 mg per day, the treatment was switched to ipilimumab 3 mg/kg plus nivolumab 1 mg/kg. After two treatments, the patient was admitted to the Oncology ward due to high body temperature, neck stiffness, headache, and photosensitivity. The patient had increased serum S100B concentration (1⋅2 μg/L). Analysis of cerebrospinal fluid showed lymphocytosis and elevated CXCL10. Brain MRI showed partial response of brain metastases. Simultaneously the patient was diagnosed with grade three hepatitis. The patient was started on prednisolone 100 mg daily and his meningitis-like symptoms gradually improved over the following 5 days. S100B and NfL dynamics are shown in Fig. 1a and Fig. 2a. S100B peaked two days before NFL and S100B normalized fast whereas NfL normalized more slowly.

***Patient 8*** *–* A patient in their sixties with symptoms of headache and nausea was diagnosed with multiple brain metastasis of malignant melanoma. The patient started treatment with cortisone and BRAF and MEK-inhibitor. After six months the cortisone was tapered, and the treatment was switched to ipilimumab 3 mg/kg plus nivolumab 1 mg/kg. Within a few days the patient starts getting headaches and a numb sensation in the fingers. The symptoms progress over the following ten days, suffering from temperatures above 104 F, severe fatigue, confusion, and aphasia. Serum S100B was increased (0⋅14 mg/L). Brain MRI show a very slight increase in size in a couple of metastases. Infectious causes were excluded. Inflammation in central nervous system was confirmed by lymphocytosis and elevated levels of CXCL13 and brain damage marker Tau in cerebrospinal fluid. Simultaneously the liver enzymes were elevated indicating hepatitis grade one. The patient’s condition improved over the following three days on high dose cortisone (methylprednisolone 1 mg/kg intravenously) with fatigue and short-term memory loss as only remaining symptoms. Cortisone was tapered slowly, and the patient was restarted on BRAF plus MEK-inhibitor. S100B and NfL dynamics is shown in Fig. 3f. S100B had an earlier peak than NfL and normalized faster.

***Patient 9*** *–*  A patient in their sixties was diagnosed with multiple liver metastases originating from a uveal melanoma. The patient was included in a clinical trial (Scandium II) and underwent an isolated hyperthermic liver perfusion followed by four doses of ipilimumab 3 mg/kg plus nivolumab 1 mg/kg. Five days after the fourth treatment the patient suddenly developed severe fatigue, fever, confusion, and dizziness. The patient's condition worsened during the following week, feeling more disoriented and needing assistance to walk due to impaired balance. Simultaneously the patient had a hepatitis grade one. Serum S100B was increased (0⋅17 μg/L). The patient was admitted to the Oncology ward and started treatment with high dose cortisone (methylprednisolone 1⋅3 mg/kg). The following day (after 2 doses of intravenous cortisone) a lumbar puncture was performed that show no signs of infection, brain damage markers were normal but lymphocytes, CXCL10 and CXCL13 were elevated, confirming inflammation of the central nervous system. Brain MRI was normal. At this point the patient’s condition was already much improved and after just two days of high dose cortisone the patient was fully recovered. S100B and NfL dynamics are shown in Fig. 3g. The patient had a distinct peak in serum S100B, but plasma NfL remained normal.

**SUPPLEMENTARY FIGURES**

**
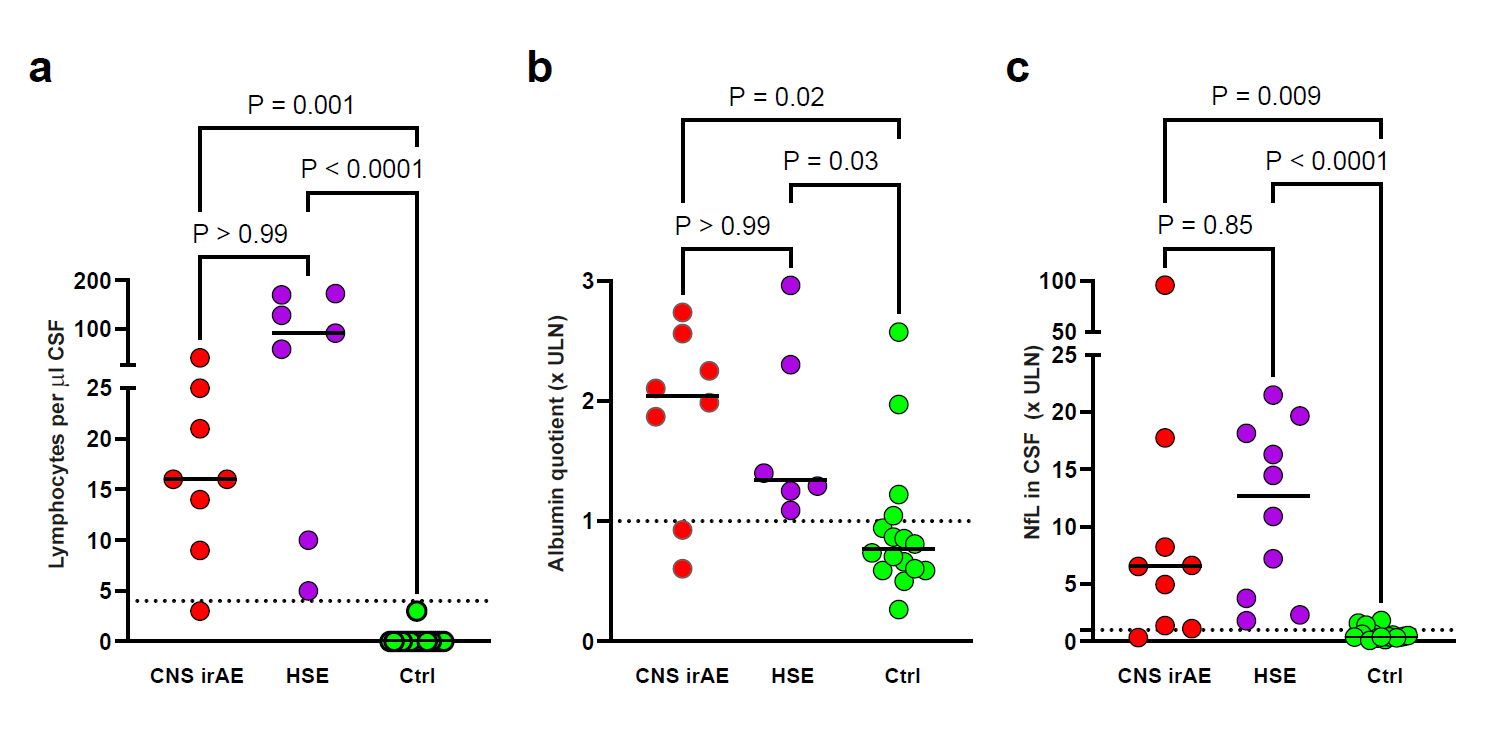
**

**Figure S1. Lymphocytes, albumin quotient and NfL in cerebrospinal fluid from CNS irAE patients, herpes simplex virus encephalitis patients and SLE controls**

(a) Lymphocytes in cerebrospinal fluid from CNS irAE patients (n=8), Herpes simplex virus encephalitis patients (HSE) (n=7) and SLE patients without CNS inflammation (Ctrls) (n=16). Dotted line indicates upper limit of normal (= 4 lymphocytes/ ml CSF). Horizontal lines indicate median values. (b) Albumin quotient (albumin in CSF/albumin in serum) in CNS irAE patient's (n=8), HSE (n=6) and Ctrls (n=16). Dotted line indicates age adjusted upper limit of normal (6⋅8 in patients below 45 years; 10⋅2 in patients above 45 years). Horizontal lines indicate median values. (c) NfL in CSF from CNS irAE (n=9) , HSE (n=10) and Ctrls (n=16). Dotted line indicates age-adjusted upper limit of normal (age <30 years < 380 ng/L; 30 – 39 years < 560 ng/L; 40-59 years < 890 ng/L; >59 years < 1850 ng/L). CNS irAE patients were compared to both HSE and Ctrls (Kruskal-Wallis test; p<0⋅05 was considered significant) and p-values are indicated in the graphs. Horizontal lines indicate median values.


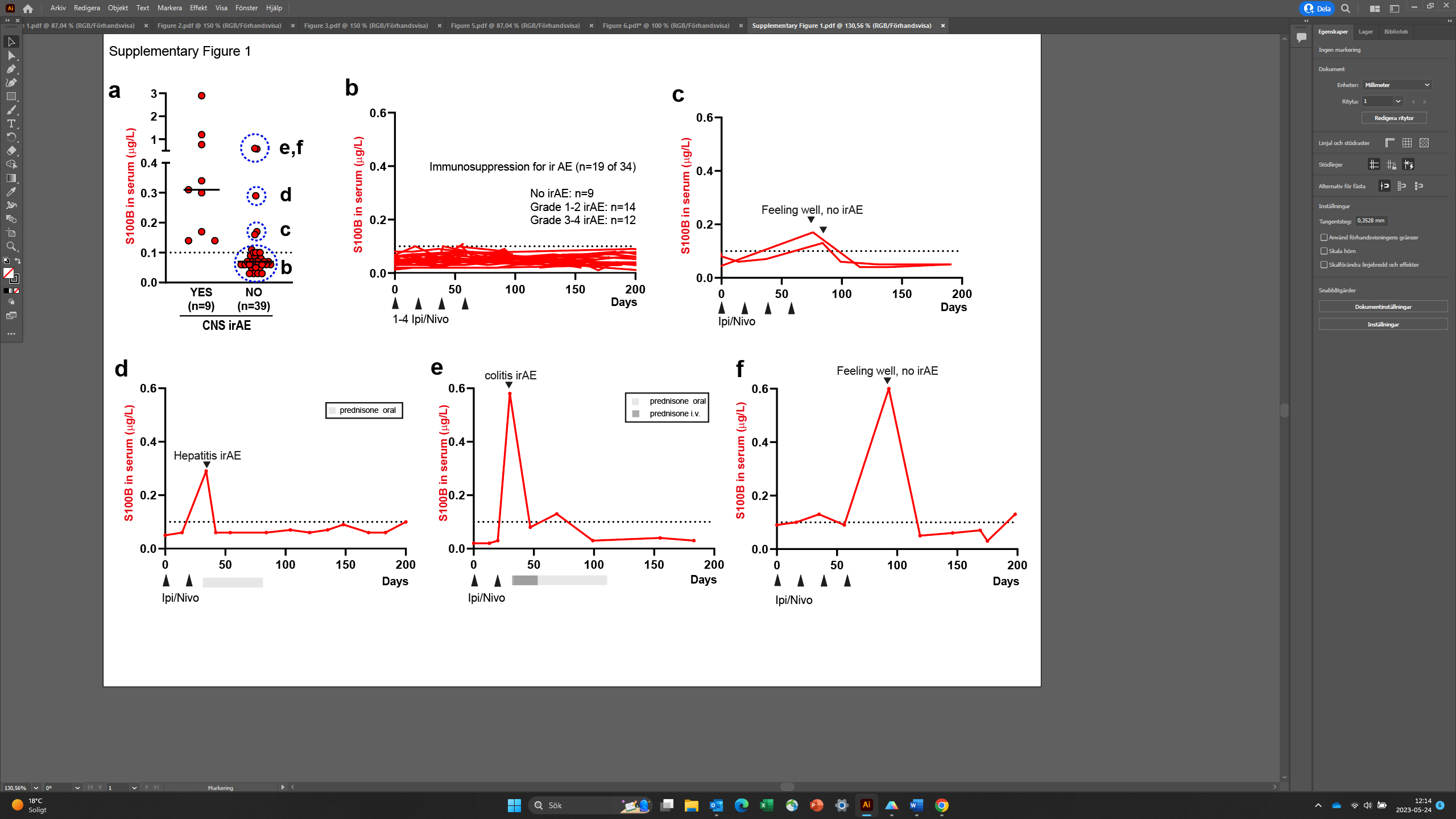


**Figure S2. Serum S100B dynamics in ipi+nivo treated patients without CNS irAE**

(a) Peak values of S100B in serum in patients without CNS irAE (n=39); the S100B did not exceed the upper limit of normal in 34 patients (encircled, b), 2 had slightly increased S100B (encircled, c) and 3 had S100B values more than twice the upper limit of normal (encircled, d,e,f). Horizontal lines indicate median values. (b) S100B concentrations over time (up to 200 days) in patients who never exceeded the normal range (n=34). (c) S100B in the two patients with isolated minor increase in S100B. None of the patients reported any irAE or symptoms at that time. (d) Peak in serum S100B concentration in patient after the second ipi+nivo. At that time the patient had an asymptomatic isolated increase in AST and ALT (grade II irAE hepatitis) and was treated with oral prednisone. (e) Peak in serum S100B concentration in patient after 2 ipi+nivo treatments. At that time point the patient was hospitalized due to grade IV colitis (without any neurological symptoms) and treated with intravenous methylprednisolone. S100B normalized and the patient improved. (f) Peak in serum S100B 4 weeks after the fourth dose of ipi+nivo. The patient had no symptoms and was not treated with any immunosuppression.

**
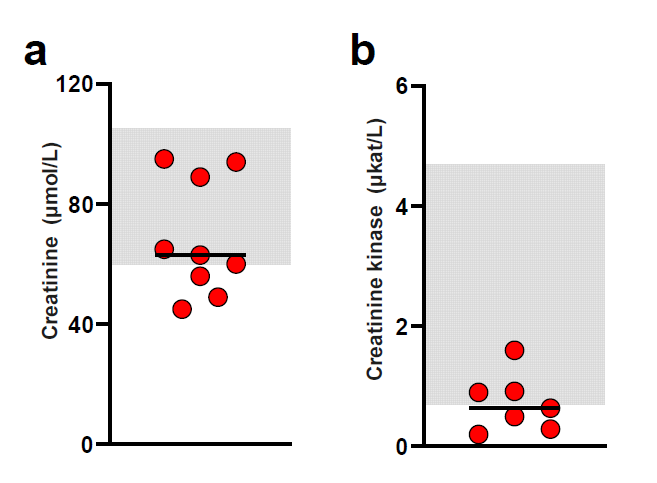
**

**Figure S3. Creatinine and creatinine kinase in patients with CNS irAE**

(a) Blood levels of creatinine was normal in six out of nine patients during CNS irAE and slightly below normal in three patients. (b) Creatinine kinase in blood was analysed in seven patients during CNS irAE and was normal in three patients and below normal in four patients. Horizontal lines indicate median (a and b).

**
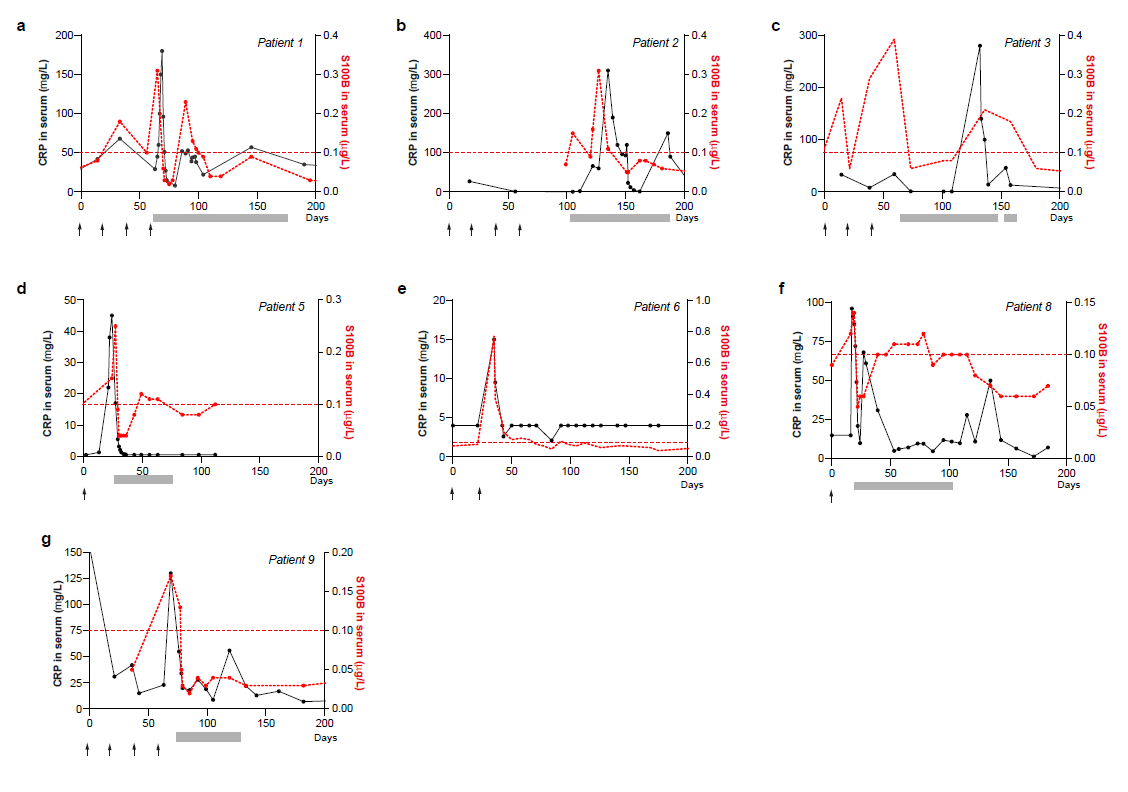
Figure S4. Covariation of S100B and CRP in patients with CNS irAE**

(a-g) Serum CRP (black curve, left y-axis) and S100B concentrations (dotted red curve, right y-axis) in CNS irAE patients during the first 200 days (x-axis) following the first treatment with ipilimumab and nivolumab (time for ipi + nivo treatments indicated with arrows under the x-axis). Period of immunosuppression is indicated by grey box under the x-axis. The upper limit of normal for S100B is indicated with a dotted red line. The patient numbers refer to Table 1 and case descriptions in the Data Supplement.

**
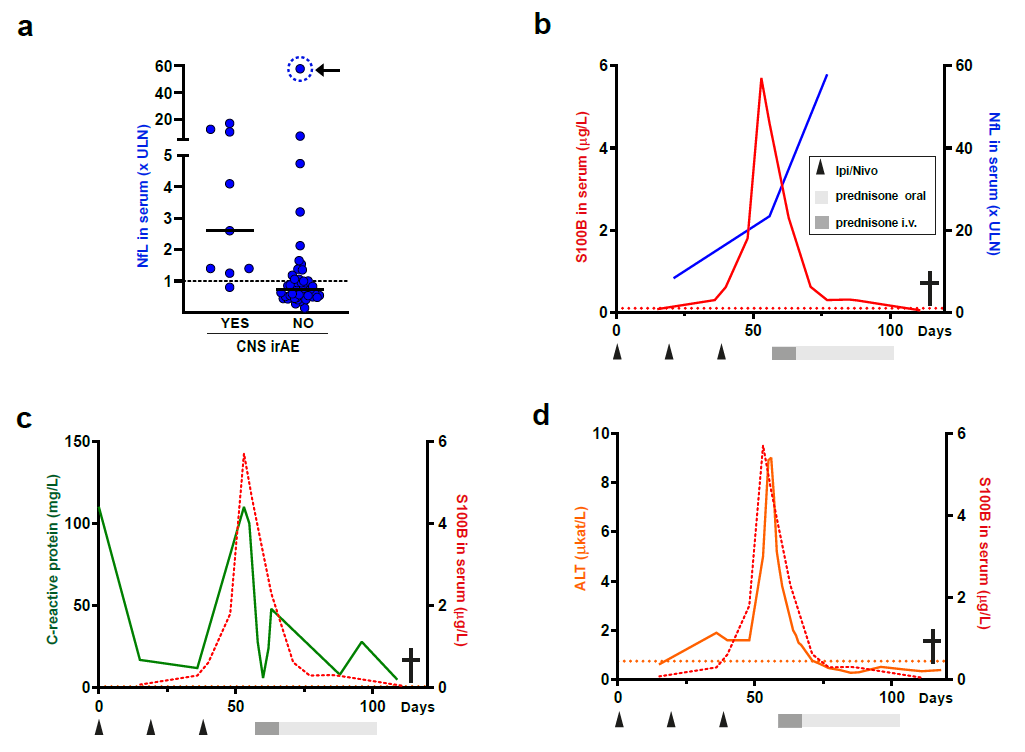
Figure S5. Patient with rapid increase in NfL following ipi + nivo treatment**

(a) One patient without CNS irAE diagnosis had an extremely high plasma NfL concentration (encircled, arrow) 77 days after receiving the first of three ipi + nivo treatments. Horizontal lines indicate median values. (b) S100B (red curve, left y-axis) gradually increased after treatment start and peaked 53 days after the first ipi + nivo. There is no baseline NfL, but 21 days after the first ipi + nivo NfL was increased to 8⋅3 times the upper limit of normal (ULN) (blue curve, right y-axis). At day 56, NfL was further increased to 23⋅4 times ULN and at day 77 to 57⋅9 times ULN. CRP (c) peaked at the same day as S100B (day 53), and ALT (d) peaked at day 56. At this point intravenous methylprednisolone was started (due to grade 3 hepatitis). The patient experienced fever and extreme fatigue. The patient improved clinically but a few weeks later rapidly deteriorated again and died (cross). Dotted lines indicate upper normal limit of S100B (a, 0⋅1 μg/L), CRP (b, 4 mg/L ) and ALT (d, 0⋅75 μkat/L). Administration of corticosteroids is indicated in grey below the x-axis.

**
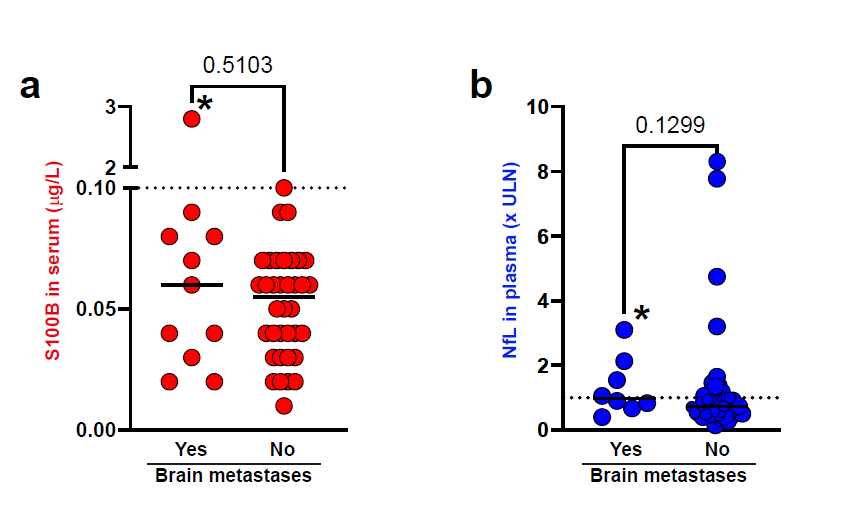
Figure S****6. Concentrations of S100B and NfL in blood in patients with or without brain metastases**

(a) Serum S100 concentrations in patients with (n=11= or without (n=36) brain metastases. The samples were taken at baseline, before ipi+nivo treatment. Horizontal lines indicate median values. (b) Plasma NfL concentration in patients with (n=8) or without (n=17) brain metastases. Asterisk (*) indicates patient with S100B positive melanoma with extensive intra and extracranial metastases. The S100B and NfL dynamics over time in this patient is shown in Fig 1b and Fig 2b. Horizontal lines indicate median values. The p-values were determined with the Mann-Whitney U test .

**SUPPLEMENTARY Tables**

**Table S1. S100B cohort – ipi+nivo treated patients without CNS irAE**

| **Number** | **Age** | **Diagnosis** | **Brain metastasis** | **# of ipi+nivo (dose)** | **irAE (CTCAE grade)** | **irAE treatment** |
| --- | --- | --- | --- | --- | --- | --- |
| **1** | 37 | CMM | Yes | 2 (ipi3+nivo1) | Colitis (3), Arthralgia (3) | CORT, MTX |
| **2** | 54 | CMM | No | 3 (ipi3+nivo1) | Colitis (2) | CORT |
| **3** | 50 | CMM | No | 4 (ipi1+nivo3) | Myalgia (1) | none |
| **4** | 71 | CMM | Yes | 1 (ipi3+nivo1) | Colitis (4) | CORT, IFX |
| **5** | 54 | CMM | No | 2 (ipi1+nivo3) | Pneumonitis (2) | CORT |
| **6** | 66 | CMM | Yes | 1 (ipi3+nivo1) | none |  |
| **7** | 72 | CMM | No | 2 (ipi3+nivo1) | Hepatitis (3), Myalgia (1) | CORT |
| **8** | 71 | CMM | No | 4 (ipi1+nivo3) | Arthralgia (3) | CORT |
| **9** | 60 | CMM | Yes** | 4 (ipi1+nivo3) | Hypophysitis | CORT |
| **10** | 51 | CMM | Yes** | 3 (ipi3+nivo1) | Hypophysitis | CORT |
| **11** | 56 | CMM | No | 4 (ipi1+nivo3) | Dermatitis (2) | TST |
| **12** | 75 | CMM | No | 4 (ipi1+nivo3) | none |  |
| **13** | 55 | UM | No | 4 (ipi3+nivo1) | none |  |
| **14** | 65 | UM | No | 2 (ipi3+nivo1) | Arthralgia (3), Hepatitis (3) | CORT |
| **15** | 59 | CMM | No | 2 (ipi3+nivo1) | Colitis (4), Pneumonitis (2) | CORT |
| **16** | 59 | CMM | No | 1 (ipi1+nivo3) | Colitis (3) Pneumonitis (2) | CORT, IFX |
| **17** | 79 | CMM | No | 4 (ipi1+nivo3) | Thyroiditis | none |
| **18** | 69 | CMM | No | 2 (ipi1+nivo3) | Gastritis (3) Hepatitis (2) | CORT, MMF |
| **19** | 72 | MM | No | 4 (ipi1+nivo3) | Colitis (3), Thyroiditis | CORT |
| **20** | 73 | UM | No | 2 (ipi3+nivo1) | Colitis (3) Pneumonitis (2) | CORT, IFX |
| **21** | 81 | CMM | No | 4 (ipi1+nivo3) | Hepatitis (1) Thyroiditis | none |
| **22** | 69 | CMM | No | 4 (ipi1+nivo3) | Hypophysitis | CORT |
| **23** | 62 | CMM | Yes | 1 (ipi3+nivo1) | Thyroiditis | none |
| **24** | 62 | CMM | No | 4 (ipi1+nivo3) | Hepatitis (1) | none |
| **25** | 77 | CMM | Yes | 4 (ipi3+nivo1) | none |  |
| **26** | 49 | CMM | No | 4 (ipi1+nivo3) | none |  |
| **27** | 79 | CMM | No | 4 (ipi1+nivo3) | none |  |
| **28** | 60 | CMM | No | 4 (ipi1+nivo3) | none |  |
| **29** | 47 | RCC | No | 3 (ipi1+nivo3) | Dermatitis (3) | CORT |
| **30** | 80 | RCC | No | 2 (ipi1+nivo3) | Myalgia (2) | CORT |
| **31** | 59 | RCC | No | 2 (ipi1+nivo3) | none |  |
| **32** | 55 | RCC | No | 4 (ipi1+nivo3) | none |  |
| **33** | 63 | RCC | No | 4 (ipi1+nivo3) | Thyroiditis | none |
| **34** | 74 | RCC | No | 4 (ipi1+nivo3) | Papilledema (2) | CORT |
| **35** | 71 | RCC | No | 4 (ipi1+nivo3) | Artralgia (2), dermatitis (2) | CORT |
| **36** | 65 | RCC | No | 4 (ipi1+nivo3) | none |  |
| **37** | 60 | RCC | No | 4 (ipi1+nivo3) | Colitis (3) | CORT, IFX |
| **38** | 70 | RCC | No | 4 (ipi1+nivo3) | none |  |
| **39** | 67 | CMM | No | 3 (ipi1+nivo3) | none |  |

*Patient described in detail in PMID: 34215689.

**Treated (surgery/radiation)

CMM, cutaneous malignant melanoma; RCC, renal cell carcinoma; UM, uveal melanoma; MM, mucosal melanoma; ipi3+nivo1, ipilimumab 3mg/kg + nivolumab 1 mg/kg; ipi1+nivo3, ipilimumab 1 mg/kg + nivolumab 3 mg/kg; irAE, immune related adverse events; CTCAE, common criteria for adverse events; CORT, corticosteroids; MMF, Mycophenolate mofetil; IVIG, intravenous immunoglobulins; CF, cyclophosphamide; IFX, infliximab; CRT, corticosteroid replacement therapy; TST, topical steroid treatment; IL6i, IL6 inhibitor

**Table S2. NfL cohort – ipi+nivo and single nivo treated patients without CNS irAE**

| **Number** | **Age** | **Brain metastasis** | **Treatment** | **irAE (CTCAE grade)** | **irAE treatment** |
| --- | --- | --- | --- | --- | --- |
| **1** | 73 | Yes* | pembro | Colitis (3) | CORT, IFX |
| **2** | 42 | No | ipi3+nivo1 | Dermatomyositis (3) | CORT, MTX |
| **3** | 56 | No | nivo | Polyarthritis (3) | CORT, MTX, IFX |
| **4** | 73 | No | pembro | Sarcoidosis, neutropenia | CORT, G-CSF |
| **5** | 75 | No | nivo | Polyarthritis | CORT |
| **6** | 81 | Yes* | pembro | None |  |
| **7** | 63 | Yes | nivo | None |  |
| **8** | 69 | No | nivo | Hypophysitis | CRT |
| **9** | 79 | No | ipi1+nivo3 | Thyreoditis, vitiligo | THRT |
| **10** | 39 | Yes | ipi3+nivo1 | Colitis (3), Polyarthritis (3) | CORT, MTX, IFX |
| **11** | 71 | No | ipi1+nivo3 | None |  |
| **12** | 77 | No | pembro | None |  |
| **13** | 81 | No | nivo | Colitis (1) Sarcoidosis | CORT |
| **14** | 53 | No | ipi3+nivo1 | Hepatitis (4) | CORT, MMF |
| **15** | 67 | No | nivo | None |  |
| **16** | 89 | No | nivo | Thyreoditis, dermatitis | CORT, THRT |
| **17** | 84 | No | nivo | Colitis (3) | CORT |
| **18** | 51 | No | nivo | None |  |
| **19** | 75 | No | nivo | None |  |
| **20** | 58 | No | nivo | Thyreoditis | THRT |
| **21** | 60 | No | ipi3+nivo1 | Polyarthritis (3) | CORT |
| **22** | 81 | No | pembro | Thyreoditis, | THRT |
| **23** | 66 | No | pembro | None |  |
| **24** | 83 | No | pembro | None |  |
| **25** | 78 | No | pembro | Colitis (3) | CORT |
| **26** | 94 | No | nivo | Nephritis (2) | CORT |
| **27** | 80 | No | pembro | Dermatitis (3) | CORT |
| **28** | 83 | No | pembro | Nephritis (2), Artralgia (3) | CORT |
| **29** | 59 | No | pembro | None |  |
| **30** | 60 | No | pembro | None |  |
| **31** | 83 | No | nivo | Nephritis (3) | CORT |
| **32** | 75 | No | ipi1+nivo3 | Sarcoidosis, Artralgia (2) | CORT |
| **33** | 71 | No | pembro | Myalgia (2) | CORT |
| **34** | 78 | No | ipi1+nivo3 | None | none |
| **35** | 67 | No | pembro | None |  |
| **36** | 82 | No | ipi1+nivo3 | Colitis (3) | CORT |
| **37** | 75 | No | ipi1+nivo3 | Hepatitis (1) | none |
| **38** | 76 | No | nivo |  | CORT |
| **39** | 80 | No | ipi1+nivo3 | None |  |
| **40** | 74 | No | nivo | Colitis (3) | CORT |
| **41** | 61 | No | ipi1+nivo3 | Myalgia (2) | CORT |
| **42** | 74 | No | ipi1+nivo3 | Hepatitis (2) Colitis (2) | CORT |
| **43** | 76 | No | nivo | None |  |
| **44** | 55 | No | nivo | Hepatitis (3), Colitis (1) | CORT |
| **45** | 64 | No | ipi3+nivo1 | Hypophysitis, Thyroiditis | CRT, THRT |
| **46** | 58 | Yes | ipi3+nivo1 | None |  |
| **47** | 79 | No | nivo | Vitiligo |  |
| **48** | 75 | No | nivo | None |  |
| **49** | 79 | Yes | ipi3+nivo1 | Thyroiditis | THRT |

*Treated (surgery/radiation)

ipi3+nivo1, ipilimumab 3mg/kg + nivolumab 1mg/kg; ipi1+nivo3, ipilimumab 1mg/kg + nivolumab 3mg/kg; pembro, pembrolizumab; irAE, immune related adverse events; CTCAE, common criteria for adverse events; CORT, corticosteroids; MMF, Mycophenolate mofetil; MTX, methotrexate; IFX, infliximab; CRT, corticosteroid replacement therapy; THRT, Thyroid Hormone Replacement Therapy; G-CSF, Granulocyte Colony Stimulating Factor

**Table S3. Neuronal autoantibodies in blood**

| **Antibodies blood** | **Case 1*** | **Case 2** | **Case 3** | **Case 4** | **Case 5** | **Case 6** | **Case 7** | **Case 8** | **Case 9** |
| --- | --- | --- | --- | --- | --- | --- | --- | --- | --- |
| Anti-NMDA-receptor | negative | negative |  | negative | negative | negative | negative |  | negative |
| Anti-LGI1 | negative | negative |  | negative | negative | negative | negative |  | negative |
| Anti-CASPR2 | negative | negative |  | negative | negative | negative | negative |  | negative |
| Anti-GABA-B | negative | negative |  | negative | negative | negative | negative |  | negative |
| Anti-VGCC PQ-type | negative | negative |  |  |  | negative |  |  |  |
| Anti-AMPA 1/2-receptor | negative | negative |  | negative | negative | negative | negative |  | negative |
| Anti-DPPX | negative | negative |  | negative | negative | negative | negative |  | negative |
| Anti-CV2 | negative | negative | negative |  | negative | negative |  |  | negative |
| Anti-Amphiphysin | negative | negative |  |  | negative | negative |  |  | negative |
| Anti-Ma2/Ta | negative | negative | negative |  | negative | negative |  |  | negative |
| Anti-Tr | negative | negative |  |  | negative | negative |  |  | negative |
| Anti-Recoverin | negative | negative |  |  | negative | negative |  |  | negative |
| Anti SOX1 | negative | negative |  |  | negative | negative |  |  | negative |
| Anti-Zic4 | negative | negative |  |  | negative | negative |  |  | negative |
| Anti-Hu Ri Yo | negative | negative | negative | negative | negative | negative | negative |  | negative |
| Anti-Aquaporin-4 (NMO) | negative |  |  | negative | negative |  | negative |  | negative |
| Anti-PCA2 | negative | negative | negative |  |  | negative | negative |  | negative |
| Anti-MOG |  |  |  | negative | negative |  | negative |  | negative |

| **Antibodies CSF** | **Case 1*** | **Case 2** | **Case 3** | **Case 4** | **Case 5** | **Case 6** | **Case 7** | **Case 8** | **Case 9** |
| --- | --- | --- | --- | --- | --- | --- | --- | --- | --- |
| Anti-NMDA-receptor | negative | negative |  | negative | negative | negative | negative |  | negative |
| Anti-LGI1 | negative | negative |  | negative | negative | negative | negative |  | negative |
| Anti-CASPR2 | negative | negative |  | negative | negative | negative | negative |  | negative |
| Anti-GABA-B | negative | negative |  | negative | negative | negative | negative |  | negative |
| Anti-VGCC PQ-type | negative | negative |  |  |  | negative |  |  |  |
| Anti-AMPA 1/2-receptor | negative | negative |  | negative | negative | negative | negative |  | negative |
| Anti-DPPX | negative | negative |  | negative | negative | negative | negative |  | negative |
| Anti-CV2 | negative | negative | negative |  | negative | negative |  |  | negative |
| Anti-Amphiphysin | negative | negative |  |  | negative | negative |  |  | negative |
| Anti-Ma2/Ta | negative | negative | negative |  | negative | negative |  |  | negative |
| Anti-Tr | negative | negative |  |  | negative | negative |  |  | negative |
| Anti-Recoverin | negative | negative |  |  | negative | negative |  |  | negative |
| Anti SOX1 | negative | negative |  |  | negative | negative |  |  | negative |
| Anti-Zic4 | negative | negative |  |  | negative | negative |  |  | negative |
| Anti-Hu Ri Yo | negative | negative | negative | negative | negative | negative | negative |  | negative |
| Anti-Aquaporin-4 (NMO) | negative |  |  | negative | negative |  | negative |  | negative |
| Anti-PCA2 | negative | negative | negative |  |  | negative | negative |  | negative |
| Anti-MOG |  |  |  | negative | negative |  | negative |  | negative |

**Table S4. Neuronal autoantibodies in CSF**

**Table S5. Cytokines and chemokines in CSF**

| **Cytokine** | **Case 1*** | **Case 2** | **Case 3** | **Case 4** | **Case 6** | **Case 7** | **Case 9** |
| --- | --- | --- | --- | --- | --- | --- | --- |
| IL-1β (<5 pg/L) | 5⋅4 |  |  | <5 | <5 | <5 | 8⋅3 |
| IL-2 (<16 pg/L) | <16 | <16 |  | <16 | <16 |  | <16 |
| IL-4 (<16 pg/L) | <0⋅25 |  |  | <0⋅25 | <0⋅25 |  | <0⋅25 |
| IL-5 (<8 pg/L) | 3⋅9 |  |  | <3⋅9 | <3⋅9 |  | <3⋅9 |
| IL-6 (<50 pg/L) | 12 | 14 |  | **240** | 4⋅5 | 5 | 4⋅5 |
| IL-8 (<90 pg/L) | **360** | **250** |  | **650** | **92** | 78 | **440** |
| IL-10 (<20 pg/L) | 12 | 28 |  | 13 | <5 | <5 | <5 |
| IL-12 | <7⋅8 |  |  | <7⋅8 | <7⋅8 | <7⋅8 | <7⋅8 |
| IFN-γ (>8 pg/L) | 22 |  |  | 119 | <7⋅8 | <7⋅8 | <7⋅8 |
| GM-CSF (pg/L) | <7⋅8 |  |  | <7⋅8 | <7⋅8 |  | <7⋅8 |
| TNF-a (<25 pg/L) | 17 | 17 | 12 | 9⋅9 | 4⋅8 | 5 | 6⋅3 |
| CXCL10 (38-360) |  |  |  | **>5000** | **1500** | **2100** | **1500** |
| CXCL13 (<7⋅8 pg/L) |  | **1700** | **72** | **49** | **281** | <7⋅8 | **14** |
